# Investigating the roles and training of paediatric research nurses working across Europe: a questionnaire-based survey

**DOI:** 10.1136/bmjpo-2017-000170

**Published:** 2017-09-05

**Authors:** Gareth J Veal, Salma Malik, Mariangela Lupo, Susan MacFarlane, Pirkko Lepola, Mary Costello, Adriana Ceci, Carine Boué, Charlotte Lecour, Annette Otto, Maryam Rastegari, Philip Berry

**Affiliations:** 1 Northern Institute for Cancer Research, Newcastle University, Newcastle upon Tyne, UK; 2 Service de Pharmacologie Clinique, Hospices Civils de Lyon, Lyon, France; 3 Consorzio per Valutazioni Biologiche e Farmacologiche, TEDDY, Bari, Italy; 4 Scottish Children’s Research Network, Tayside Children’s Hospital, Dundee, UK; 5 Finnish Investigators Network for Pediatric Medicines, Department of Children and Adolescents, Helsinki University Hospital, Helsinki, Finland; 6 National Children’s Research Centre, Our Lady’s Children’s Hospital Crumlin, Dublin, Ireland; 7 Fondazione per la Ricerca Farmacologica Gianni Benzi Onlus, Valenzano, Italy

**Keywords:** research nurse, training, paediatrics, clinical trials

## Abstract

**Background:**

The key role played by research nurses in coordinating clinical trials in a paediatric setting has developed in line with increasing complexities of trial design. A questionnaire-based survey was conducted to investigate the training of research nurses involved in paediatric trials across Europe, to identify potential training needs and compare roles across specialties and countries.

**Methods:**

A structured, cross-sectional questionnaire survey was used, with the aim of describing and quantifying research nurse experiences. The questionnaire was designed to cover four main areas of interest: demographics, training, clinical trial experience and research nurse roles/activities.

**Results:**

The questionnaire was completed by 341 respondents across 45 different specialties in 20 European countries. A higher percentage of research nurses within 3 years of taking up post were dissatisfied with the level of training received (16%), as compared with those in post for 3–6 years (8%) and >6 years (6%). There was a trend towards a higher percentage of respondents receiving self-funded training in mainland Europe, with reported values of 15%–20%, as compared with <5% in the UK and Ireland. Only 3% of research nurses prescribed investigational medicinal products in a clinical trial setting, with contrasting roles observed between countries.

**Conclusions:**

While high levels of training satisfaction were observed, 67% of respondents felt they would benefit from additional training in line with frequently changing practices. Currently, low levels of nurse prescribing are observed in a paediatric clinical trial setting across Europe. Appropriate research nurse training programmes should be promoted through national networks across Europe.

**What is already known on this topic?**

- Research nurses are increasingly involved in all stages of the development of complex clinical trials conducted across paediatric specialties.
- It is important that research nurses are appropriately trained for the various regulatory, methodological, ethical and administrative aspects of clinical trial design.
- Few studies have been published focusing on the level of training and roles played by research nurses working in paediatric specialties across Europe.

**What this study hopes to add?**

- Approximately two-thirds of research nurses felt that they would benefit from additional training in specific areas, with a clear relationship observed between length of time in post and level of training satisfaction.
- An increased level of nurse prescribing may be beneficial in paediatric specialties, with only 3% of participants prescribed investigational medicinal products in a clinical trial setting.
- Sharing of the information generated through national research nurse networks should be used to encourage the development of paediatric research nurse training programmes.

## Introduction

There is a clear need to accelerate the development of drugs across a wide range of childhood disease specialties.^1^ In order for this to be achieved, high-quality ethical research on the safety and efficacy of medicines in children is needed. In this respect, the research nurse plays an increasingly pivotal role in the successful conduct of paediatric clinical trials.^2^

Clinical trials in children have an inherent default level of complexity relating to regulatory, methodological, ethical and administrative issues, with additional burdens commonly introduced for multinational studies.^3^ The role of the research nurse has developed in line with increasing numbers of often complicated research studies commonly built into trial design, to generate as much information as possible relating to the new treatment. Outside of the collection of blood samples for routine clinical analysis, samples are frequently requested for a range of substudies including clinical pharmacology and biomarker studies, pharmacogenomics, biobanking and cytogenetics.^4^ Clinical samples will commonly be requested at multiple time points and require the collection and recording of data and completion of clinical trial visit-associated case report forms (CRFs), often via trial-specific electronic data capture (EDC) systems. These studies are carried out alongside more routine research nurse responsibilities, including day-to-day study management, patient screening, provision of patient information sheets, appropriate collection of consent/assent and collaboration with other members of the multidisciplinary team required to ensure a positive experience for study patients.^5^

The European Network of Paediatric Research at the European Medicines Agency (Enpr-EMA) consists of a consortium of research networks, investigators and centres with expertise in performing clinical trials in children and adolescents.^6^ A working group was established by Enpr-EMA to investigate potential needs and gaps in research nurse training across specialties and countries. A questionnaire-based survey was carefully planned to compare experiences and seek the views of research nurses working in paediatric settings across Europe, in addition to generating information on the extent of involvement in clinical trial activities and the specific roles carried out by research nurses in different countries and specialties. Such an approach may allow the identification of potentially desirable training models for recommendation by Enpr-EMA at a European level.

## Materials and methods

### Questionnaire design and preparation

A structured, cross-sectional questionnaire survey, informed by previous research on nurse training and questionnaire development,^7–9^ was designed to cover four main areas of interest. The first section covered basic demographic information, including disease specialties, age of children being cared for and length of time as a research nurse. The second section focused on the level, frequency and method of training received. The third area focused on clinical trials experience with regards to involvement in different types of trials and various aspects of developing and running studies. Finally, paediatric research nurse roles and activities were investigated, from patient consent, prescribing and administration of investigational medicinal products (IMPs), through to sample collection, processing and transport.

The design incorporated fixed choice and open-ended questions and was piloted on an initial cohort of 20 paediatric research nurses to check for usability. Following minor alterations and additions to the questionnaire at this point, a final version was approved for dissemination. box 1 summarises the questions incorporated in the final questionnaire and the full questionnaire is provided as an online supplementary figure 1.

Box 1 **Summary of questions in final questionnaire (response required and follow-up questions)**

- Across what specialties do you work in paediatrics? (select all that apply; if ‘other’, please provide details)
- What age of children do you work with? (select all that apply)
- How long have you worked as a research nurse?
- How many phase I, II, III or IV clinical trials have you participated in?
- Do you feel that you have received appropriate training for the role(s) you carry out in your position?
- Do you feel that you would benefit from additional training in some aspects of your job? (if ‘yes’, please comment)
- How would you best describe describe the training that you received for your role? (select one)
- If you have received Good Clinical Practice (GCP) training, has this been generic GCP training or paediatric-specific GCP training?
- If you have received additional training, please provide further information with regards to the type of training.
- How frequently do you receive training in your current job?
- How frequently do you receive GCP certified training within your role?
- How would you best describe the training that you received when you first started in post? (select all that apply)
- How would you best describe the training updates that you receive? (select all that apply)
- Which of the following activities do you have experience of actively participating in?
- Within your role, do you participate in CTIMP (Clinical Trial of an Investigational Medicinal Product) studies?
- If ‘yes’, which of the following roles do you perform?
- If ‘yes’ to any of the above, have you received specific training for this?
- Are you involved in the following types of paediatric clinical trials? (tick all that apply)

10.1136/bmjpo-2017-000170.supp1 Supplementary file 1 sf1

### Participants and data collection

The final questionnaire was made available via an electronic link through the internet-based survey tool provider Google Forms. National and disease specialty networks of paediatric research nurses were identified through Enpr-EMA networks and the identification of appropriate European groups through internet searches. A link to the Google Forms questionnaire was sent to lead network contacts alongside a letter from Enpr-EMA, explaining the purpose and aims of the survey, for dissemination to research nurses within individual networks or groups. The questionnaire was translated into French and Spanish as requested by specific networks and was made available for a 9 month period between April and December, 2016.

### Data analysis and statistical analysis

Data entry and initial analysis were carried out using Microsoft Excel 2013 and Qlik Sense V.3.1 (Qlik International AB). Statistical analysis was carried out using the χ^2^ test as appropriate using SPSS statistical software.

## Results

### Demographics of respondents

The questionnaire was completed by 341 research nurses from 20 European countries. The respondents worked across 45 different disease specialties, with 34% working with children in a single specialty, 21% in two specialties, 14% across three or four specialties and the remaining respondents (31%) working across at least five specialties. The most common specialty areas were respiratory diseases, oncology and diabetes. Respondents spanned a wide range of experience levels, with approximately 1/3 of participants (31%) having worked as research nurses for <3 years and 38% having >6 years of experience. Table 1 provides a summary of the demographics of the research nurses who participated in the study.

**Table 1:** Demographics of research nurse respondents

Characteristic	No. of respondents	%
Evaluable responses	341	
Age of patients cared for (years)		
<1	284	83
1–3	278	82
4–10	295	87
11–16	287	84
>16	234	69
Length of time working as a research nurse (years)		
<3	106	31
3–5	90	26
6–10	81	24
>10	48	14
Unknown	16	4.7
Country		
UK	189	55
France	22	6.5
Germany	16	4.7
Norway	16	4.7
Spain	15	4.4
Ireland	14	4.1
Switzerland	13	3.8
Netherlands	11	3.2
Denmark	10	2.9
Austria	8	2.3
Finland	7	2.0
Belgium	6	1.7
Sweden	4	1.2
Portugal	3	0.9
Other*	7	2.3
Number of specialties		
1	116	34
2	73	21
3–4	46	13
5–9	84	25
10–14	18	5.3
15–20	4	1.2

*Country grouping ‘other’ includes Italy (2), Greece (2), Hungary (1), Luxembourg (1) and Turkey (1).

### Training received and satisfaction with level of training

Data collected on frequency of training suggested that research nurses received regular training, with 53% receiving formal training at 6 monthly, annual or 2 yearly intervals, and 38% being trained ‘as needed’. Less than 10% of respondents received training at intervals of >2 years.

**Figure 1:**
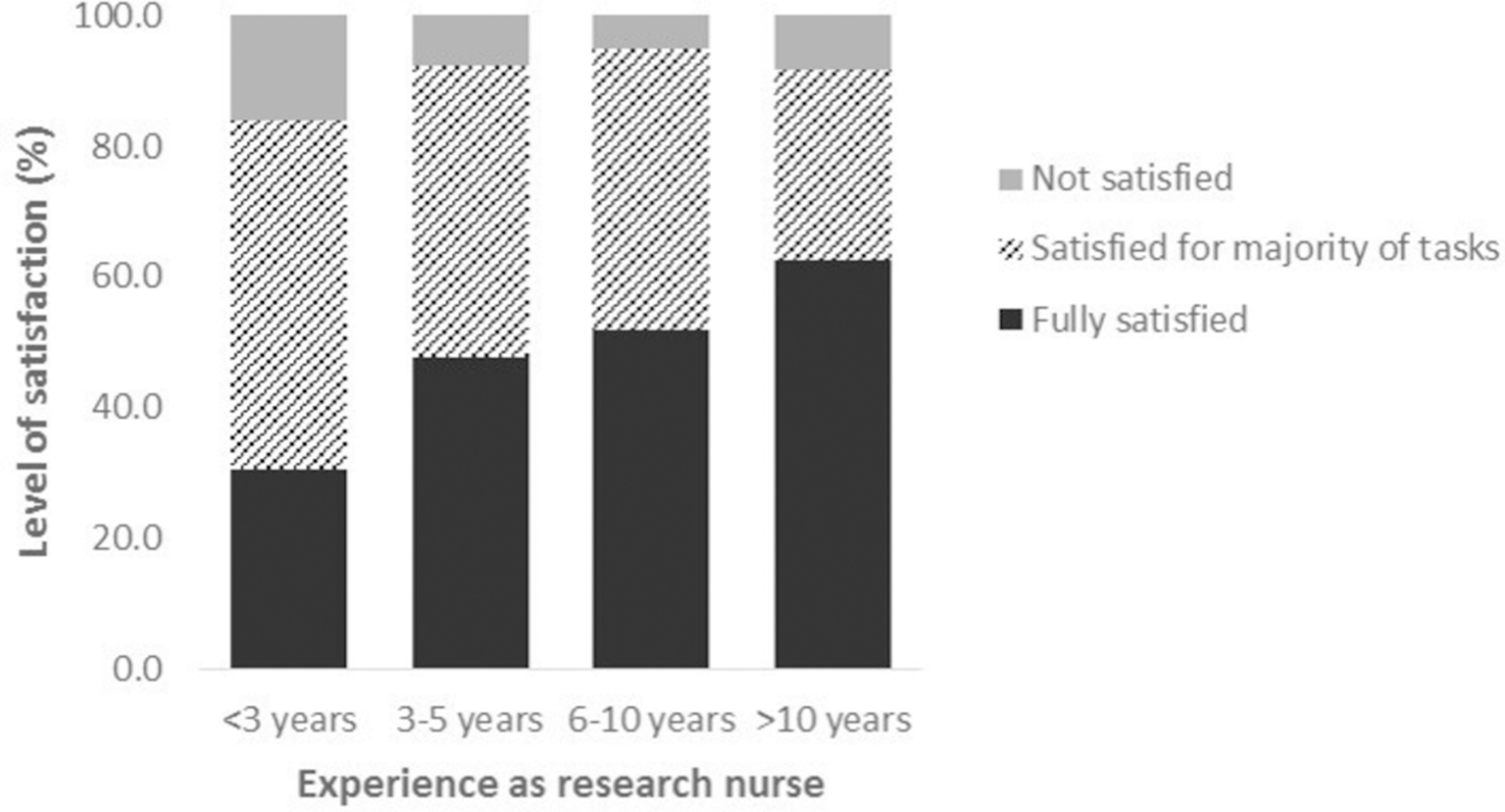
Level of satisfaction with training received by level of experience in terms of length of time in research nurse post.

A total of 147 research nurses (43%) were fully satisfied with the level of training received, with 32 (9%) respondents not satisfied and a further 145 (43%) satisfied that they were appropriately trained for the majority of tasks that they carried out; the remaining 17 (5%) participants failed to respond to this question. Further analysis suggested that a significantly higher percentage of research nurses within the first 3 years of taking up their post were dissatisfied with their level of training (16%) as compared with those with 3–6 years (8%) and >6 years (6%) of experience (p<0.001). Overall, there was a clear trend towards a relationship between length of time in post and level of training satisfaction (see figure 1). Looking at the results obtained geographically, for those countries with at least 10 respondents, higher percentages of nurses dissatisfied with the level of training received were observed in Norway (25%) and Denmark (20%). In contrast, 100% of respondents from Spain and the Netherlands were either fully satisfied or satisfied with the level of training received for the majority of tasks carried out. In response to the direct question as to whether they would benefit from extra training in some aspects of their job, 67% of all respondents indicated that this would be beneficial.

With regards to the type of training received, in terms of whether this was carried out online or in person, organised internally or run by an external organisation, institution or self-funded, there were no clear trends observed in terms of the level of training satisfaction (p>0.05). Similarly, there was no relationship between the frequency of training received and the level of satisfaction reported (p>0.05). Interestingly, 68% of research nurses received training updates online and this value was also high (54%) in terms of the initial method of training received when first in post. While there were no particularly striking findings observed when these data were analysed by country, there appeared to be a higher percentage of respondents who received self-funded training in mainland Europe, with reported values of 15%–20% in Germany, Norway, Switzerland, Denmark and the Netherlands, as compared with <1% in the UK and <4% in Ireland.

Specific questions were included in the questionnaire relating to the provision of Good Clinical Practice (GCP) training, with overall 96.5% (329/341) of research nurses having received GCP training, with 40% of these respondents (132/329) having received paediatric-specific GCP training and 60% (197/329) generic GCP training. These figures were comparable across specialties and countries.

### Additional training needs

Information relating to areas of additional training that research nurses would benefit from could be categorised into the following general areas of training: regulatory issues (21% of respondents who indicated that additional training would be beneficial), clinical trial coordination/GCP-related (20%), nursing procedures (19%), information technology (IT) based (10%), data analysis (8%) and communications skills training (6%). In the area of clinical trials in particular, a wide range of training needs were identified including areas such as research governance, trial set-up, design and coordination, costing and finance. Similarly, requests for training in a wide range of nursing procedures and laboratory skills highlight the increasing requirement for research nurses to possess a wide range of skill sets consummate with complex clinical trial designs.

### Clinical trial experience

The questionnaire explored the role of the research nurse in various aspects of developing and running paediatric clinical trials. Approximately 1/3 of respondents (36%) were involved in the development of consent forms, 27% had experience of trial submission and 32% in the development of trial CRFs. Approximately half of participants (46%) had experience of developing patient information sheets. These data were consistent across countries and specialties.

### Research nurse roles and activities

**Figure 2:**
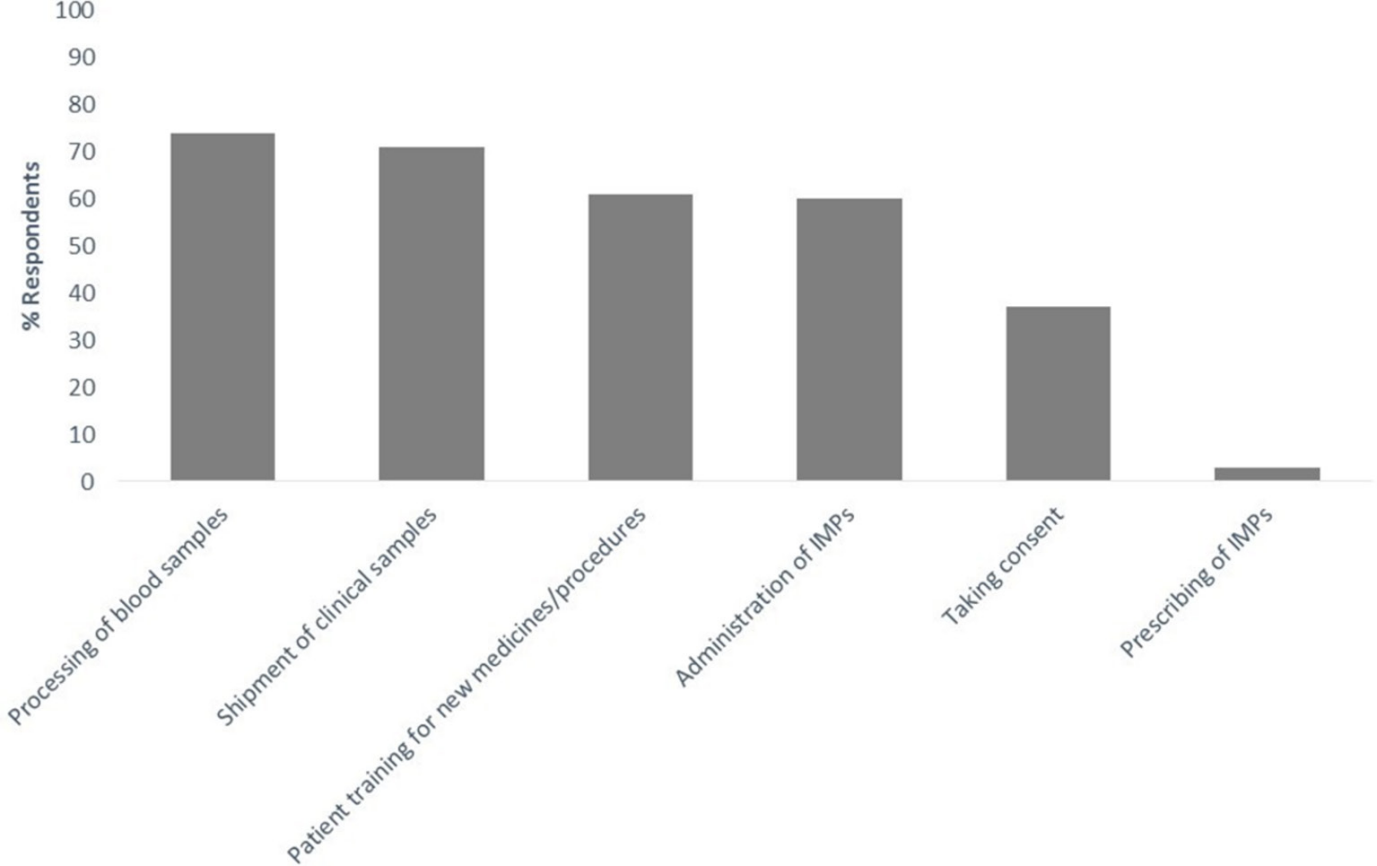
Summary of activities carried out by research nurses working in a paediatric setting. IMPs, investigational medicinal products.

In terms of the activities in which participants are commonly involved, over 70% of research nurses actively participated in the collection and processing of blood samples (252 respondents) and the shipment/transport of clinical samples (242 respondents), with approximately 60% of respondents involved in the training and education of patients in terms of the administration of new medicines or procedures (207 respondents), and the administration of IMPs (205 respondents). Approximately 1/3 of research nurses who responded to the survey were involved in taking consent and/or assent from patients (126 respondents) and only 3% (11 respondents) prescribed IMPs in a clinical trial setting (see figure 2).

Further analysis of these data by country identified wide ranges in percentages of research nurses actively involved in the defined roles and activities described above. While percentages were high across all countries for routine roles such as the collection, processing and transport of clinical samples, marked differences were seen in the percentage of respondents taking patient consent for clinical trial participation. For those countries with at least 10 respondents, less than 10% of research nurses in Germany and Spain took consent, whereas approximately half of UK research nurses (49%) and 80% of respondents from the Netherlands carried out this role. Percentages of research nurses who administered IMPs ranged from 15% in Switzerland to 87% in Spain, with small numbers of respondents (≤10%) responsible for prescribing IMPs in all countries except for Norway (13%) and Switzerland (23%). In terms of analysis of research nurse roles and activities by specialty area, there were no clear trends or differences observed.

## Discussion

The current study was carried out to gather information relating to the training and roles of research nurses who conduct paediatric clinical trials across Europe. A questionnaire-based survey was proposed and executed by Enpr-EMA, with responses gathered from 341 respondents, encompassing 45 different disease specialties and 20 European countries. As there was no explicit sampling frame, in terms of a defined list of numbers of research nurses in the networks and centres who received the questionnaire to complete, it was not possible to address the extent of non-response bias and this represents an accepted limitation of the study. Similarly, while national and disease specialty networks of paediatric research nurses were identified through Enpr-EMA networks and the identification of appropriate European groups through internet searches, many lead network contacts were not research nurses and wider circulation of the study information and link to the survey may not always have been prioritised.

Results generated from the questionnaire were generally encouraging, with 86% of respondents either fully satisfied with the level of training received, or satisfied that they were appropriately trained for the majority of tasks that they carried out. Indeed, a healthy percentage of respondents had either completed formal postbachelor education or training programmes or obtained more focused training in key areas, commonly provided by sponsors or pharmaceutical companies. However, 67% of respondents also felt that they would benefit from additional training, with a wide range of areas highlighted where training would be most beneficial. The most common areas for additional training reflected the increased complexities of modern day clinical trials and increasing requirement for research nurses to possess a wide range of skill sets.^2 3^ These included clinical trial set-up and management, IT skills, pharmacovigilance, CRF data entry and laboratory skills training. A number of respondents highlighted the challenges of keeping up to date with frequent changes to clinical trial practices relating to ever increasing GCP regulations.

In terms of how research nurses obtain training, 54% of respondents received their initial training online, when they first took up post, and 68% received training updates online. These figures highlight marked increases in online nurse training observed over the past 20 years, largely due to its convenience and flexibility. Online training can help to avoid problems relating to intensive workloads and working shifts, which could provide barriers to research nurses attending scheduled training sessions. In this respect, many studies have reported positive outcomes from online nurse training, with learning outcomes comparable or even improved as compared with face-to-face training events.^8^

One interesting point raised by several participants, related to the expectation that they would gain relevant experience through ‘on the job’ training. This theory appears to be supported by the findings of the current study, with a clear relationship observed between length of time in post and level of training satisfaction reported. Several respondents highlighted the fact that they would have benefited from more training when they first started in post, at which time they were unaware of the training opportunities available. This may be particularly relevant to countries where higher levels of dissatisfaction were reported in terms of training received. For example, in some Nordic countries, more accessible research nurse training programmes have only relatively recently been developed, following studies highlighting a need for more relevant training to be made available.^10 11^ This represents an area that could be improved, through advertising and promotion of research nurse training events. The availability of induction packs for new research nurses, containing relevant information and useful links to networks where training is available, is commonplace in some countries and should be encouraged more widely. It is accepted that the role of the research nurse may differ significantly between countries and that the current survey, while relatively expansive in terms of the number of respondents and countries involved, did not include respondents from many other European countries.

Data generated from the current study highlight the integral role that research nurses play in the running of clinical trials in paediatric specialties,^2 3^ with significant numbers of participants being involved in various wide-ranging tasks. In terms of the practicalities of working in a clinical trial setting, high percentages of respondents were involved in taking patient consent, collection and processing of samples, patient training and the administration of IMPs. Despite relatively small numbers of respondents from individual countries, apparent differences were observed in the level of involvement in activities including administration of IMPs and taking consent. Such differences may reflect guidelines and philosophies within countries, with the role of taking consent being actively encouraged in the UK, but not being seen as an appropriate research nurse role in some European countries. Indeed, it is entirely feasible that while research nurses may feel comfortable in explaining clinical trials to patients and families, they may be less amenable to being responsible for the signing of consent forms. In this respect, it is important to understand that the appropriateness of research nurses taking on this responsibility may be related to the complexity of the particular clinical trial and the IMP involved. For research nurses working in environments where roles such as the taking of consent are encouraged, competency tools are commonly available to promote patient safety and may be included in research nurse induction packs referred to above.^12^

A key role absent from the activities of the vast majority of respondents related to the prescribing of IMPs, with only 3% of research nurses performing this role. For research nurses to prescribe medicines, including unlicensed and clinical trial drugs, they are required to have the appropriate qualifications.^13^ In the UK, registered nurses with a minimum of 3 years of clinical experience must undertake a recognised ‘Nursing and Midwife Council’ accredited prescribing course through a UK university. Other countries have their own research nurse qualifications, which may contrast in terms of the level of training involved and the prescribing responsibilities of the research nurse.^14 15^

While nurse prescribing in the UK and Europe has developed significantly over the past decade, with an estimated 19 000 nurse prescribers registered in the UK in 2014,^16^ this remains a remarkably underdeveloped area. Indeed, experience in the UK indicates that differences exist between individual centres and health authorities, in terms of whether or not research nurses are able to prescribe IMPs, even if the appropriate level of training is in place. Therefore, the reported low numbers of research nurses responsible for prescribing IMPs may reflect local legislation, as opposed to a lack of desire for nurses to be involved in this area. Benefits of research nurse prescribing in the UK have been widely reported,^17 18^ and may represent an area where improvements could be made to the efficiency of running paediatric clinical trials. An increase in level of nurse prescribing would seem to represent a sensible way to optimise the skills and expertise of all health professionals working in increasingly stretched healthcare systems.^19^ It is unclear whether or not the observed increased research nurse prescribing rates in countries such as Norway and Switzerland reflects a real difference, possibly related to more accessible training and accreditation in these countries, as the numbers of respondents from the majority of countries was small. Similarly, differences highlighted between countries in the percentages of research nurses actively involved in taking patient consent and the administration of IMPs should be interpreted with caution.

## Conclusion

In summary, the study provides a useful overview of the current training status of research nurses working in paediatric medicine, highlighting potential training needs and summarising the roles and activities of research nurses across Europe. As higher percentages of respondents received self-funded training and/or were not satisfied with the level of training in some European countries, this would suggest that different training opportunities and historical working cultures may currently exist. While the level of training and general satisfaction levels expressed by research nurses is encouraging, approximately two-thirds of respondents felt that they would benefit from additional training, with commonly requested areas for further training highlighted. Increased availability and provision of research nurse training in these areas may facilitate an increased efficiency in the running of clinical trials in a paediatric setting. Sharing of the information generated in the current study through Enpr-EMA and national research nurse networks will be strongly encouraged, with a view to supporting, facilitating and developing new research training programme for paediatric research nurses across Europe. In this respect, Enpr-EMA should look to enhance the design of the European paediatric research nurse core curriculum, together with relevant European Nursing Associations, which could be then adopted across EU countries. Increased collaboration and discussion between key stakeholders will help to harmonise approaches to training and standardise the way that paediatric clinical trials are conducted across Europe, promoting improved ethical and clinical standards and the generation of robust results from clinical trials.
