# Supplementary material for: Investigating the roles and training of paediatric research nurses working across Europe: a questionnaire-based survey
Source: BMJ Paediatr Open. 2017 Sep 5;1(1):e000170. doi: 10.1136/bmjpo-2017-000170 (PMC5862212; doi:10.1136/bmjpo-2017-000170)
Supplement: Supplementary file 1 [file bmjpo-2017-000170supp001.pdf]

Survey of Research Nurse Training and Experience in a Paediatric Clinical Trial Setting

The following survey has been generated by the European Network of Paediatric Research at the European Medicines Agency (EnprEMA). A key objective of the EnprEMA network is the facilitation of studies to increase the availability of medicinal products authorised for use in the paediatric population. Due to the critical role that research nurses play in facilitating clinical trials in children, EnprEMA is carrying out this survey relating to the experiences, training and needs of research nurses working in a paediatric clinical trial setting. We are looking to collect information from as many European countries and as many different specialties as possible and would be very grateful for your assistance in this task. The survey is anonymous and should only take 5-10 minutes of your time. Further information about EnprEMA can be found through the European Medicines Agency website (http://www.ema.europa.eu/ema/).

1. Across what specialties do you work in paediatrics? (select all that apply)

Tick all that apply.

- [ ] Allergy & Immunology
- [ ] Cardiology
- [ ] Child and Adolescent Mental Health
- [ ] Dermatology
- [ ] Diabetes
- [ ] Endocrinology (including diabetes)
- [ ] Gastroenterology
- [ ] Genetics
- [ ] Haematology
- [ ] Infectious Diseases
- [ ] Neurology
- [ ] Neonatal
- [ ] Oncology
- [ ] Psychology
- [ ] Respiratory
- [ ] Rheumatology
- [ ] Other

If 'other' please provide details

____

2. What age of children do you work with? (select all that apply)
Tick all that apply.

- [ ] <1 year
- [ ] 1-3 years
- [ ] 4-10 years
- [ ] 11-16 years
- [ ] >16 years

3. How long have you worked as a research nurse?
Mark only one oval.

- ( ) <3 years
- ( ) 3-5 years
- ( ) 6-10 years
- ( ) >10 years

4. How many Phase I, II, III or IV clinical trials have you participated in?
Mark only one oval.

- ( ) 0
- ( ) 1-5
- ( ) 6-10
- ( ) >10

5. Do you feel that you have received appropriate training for the role(s) you carry out in your position?
Mark only one oval.

- ( ) Yes
- ( ) No
- ( ) For the majority of tasks that I carry out

6. Do you feel that you would benefit from additional training in some aspects of your job?
Mark only one oval.

- ( ) Yes
- ( ) No

If 'yes' please comment

____

7. How would you best describe the training that you have received for your role (select one)?

Mark only one oval.

- Non-GCP
- Generic GCP certified only
- GCP certified with supervised consent practice
- GCP with supervised consent practice and additional training
- GCP with supervised consent practice, additional training and paediatric specific GCP component

8. If you have received GCP training has this been generic GCP training or paediatric specific GCP training?

Mark only one oval.

- Generic GCP
- Paediatric specific GCP

9. If you have received additional training, please provide further information with regards to the type of training (e.g. IT skills, Information Governance, Data Protection, English, etc)

10. How frequently do you receive training in your current job?

Mark only one oval.

- At least every 6 months
- Annually
- Every 2 years
- Every 3 years or more
- Never
- As needed

11. How frequently do you receive GCP certified training within your role?
Mark only one oval.

- ◯ At least every 6 months
- ◯ Annually
- ◯ Every 2 years
- ◯ Every 3 years or more
- ◯ As stipulated by pharmaceutical companies or other authorities
- ◯ Never

12. How would you best describe the training that you received when you first started in post? (select all that apply)
Tick all that apply.

- [ ] Training course attended in person
- [ ] On-line training
- [ ] Inter-departmental training
- [ ] R&D training
- [ ] External training course
- [ ] Self-funded
- [ ] Funded by employee
- [ ] Organised by employee
- [ ] Organised or stipulated by pharmaceutical company or other relevant authority

13. How would you best describe the training updates that you receive? (select all that apply)
Tick all that apply.

- [ ] Training course attended in person
- [ ] On-line training
- [ ] Inter-department training
- [ ] R&D training
- [ ] External training course

14. Which of the following activities do you have experience of actively participating in?
Tick all that apply.

- [ ] Applications to Ethics Committees or other regulatory authorities
- [ ] Development of Patient Information Sheets
- [ ] Development of Consent Forms
- [ ] Submission of trial amendments
- [ ] Development of trial CRFs

15. Within your role do you participate in CTIMP (Clinical Trial of an Investigational Medicinal Product) studies?

Mark only one oval.

- ◯ Yes
- ◯ No

16. If yes, which of the following roles do you perform?

Tick all that apply.

- ☐ Collection and/or processing of blood samples
- ☐ Coordination of shipment/transport of clinical samples
- ☐ Taking consent
- ☐ Prescribing of IMPs (Investigational Medicinal Products)
- ☐ Administration of IMPs (Investigational Medicinal Products)
- ☐ Training/education for patients in cases of new medicines or procedures

17. If yes to any of the above, have you received specific training for this?

Mark only one oval.

- ◯ Yes
- ◯ No
- ◯ Some but not all

18. Are you involved in the following types of paediatric clinical trials (tick all that apply)?

Tick all that apply.

- ☐ National clinical trials
- ☐ European clinical trials
- ☐ International clinical trials
- ☐ Phase I/II trials
- ☐ Phase III trials
- ☐ Phase IV trials

19. In which country do you work?

____

We would like to thank you for taking the time to complete this questionnaire.
